# Navigating Dilemmas Arising from Advocacy and Resistance in Medical Education and Medical Practice

**DOI:** 10.5334/pme.1619

**Published:** 2025-02-28

**Authors:** Rachel H. Ellaway, Tasha Wyatt, Maria Hubinette

**Affiliations:** 1 Cumming School of Medicine, University of Calgary, Calgary, Alberta, Canada; 2 Center for Health Professions Education, Uniformed Services University of the Health Sciences, Bethesda, MD, USA; 3 Department of Family Practice and Centre for Health Education Scholarship, Faculty of Medicine, University of British Columbia, Vancouver, BC, Canada

## Abstract

**Background:**

Advocacy and resistance are undertheorized in medical education, yet trainees are often encouraged by their teachers to engage in these activities as a way of helping patients, mitigating healthcare system weaknesses, or challenging harms or injustices. How health professionals can and should engage in advocacy or resistance (which can be treated as a dyad of advocacy-resistance) is undertheorized, which can create confusion for trainees and lead to harms. Although acts of advocacy-resistance are often framed as pro-social, applications of advocacy-resistance can create inequity in seeking to reduce it, and they can create challenges for those trying to negotiate this perilous landscape.

**Method:**

The authors respond to the need for a more robust theoretical grounding in this space by taking a dialogical approach (based on abductive group discussion and debate, reading and rereading the literature, and collaborative writing and theory building) to explore ethical dilemmas that can arise from healthcare practitioner and trainee engagement in acts of advocacy-resistance.

**Findings:**

Four broad dilemmas arising from healthcare practitioner and trainee acts of advocacy-resistance are described: where the loci of responsibilities lie, how professional identity and agency are situated within a collective, balancing competing needs and priorities, and managing harm that can result from engaging in advocacy-resistance. The authors describe contributing factors including equity, identity, needs, priorities, responsibilities, and the advocacy-resistance dyad itself.

**Conclusions:**

In better understanding the dilemmas that acts of advocacy-resistance can create, healthcare providers, educators, and trainees should be better able to negotiate this complex and yet necessary space.

## Introduction

Advocacy in medicine can mean different things. In some contexts, a medical advocate is a layperson (such as a family member or friend) who acts as an adviser to patients and facilitates their interactions with the healthcare system. In other contexts, it is medical practitioners who are expected or encouraged to act as advocates (variously) for their patients, for the health of their communities, for their trainees, and even for their profession. However, quite what this means in training and practice is ill-defined, which makes advocacy a problematic concept in healthcare and health professions education [1] particularly as it can lead to disruption or harm (such as rule-bending or rule-breaking that leads to medical errors) and may even be pursued for unethical ends (such as helping family members or friends but not others) [2].

Resistance is also a challenging concept in the healthcare context, not least because it is considered by some to be something that healthcare providers should never engage in [3]. Despite this, much resistance in the healthcare context reflects altruistic goals, such as countering injustice and inequity. Indeed, despite their rhetorical differences, acts that are seen as advocacy by some could be considered resistance by others and vice versa [4]. Rather than approaching advocacy and resistance as distinct concepts, we treat them here as a bound dyad of advocacy-resistance [4]. This dyad allows us to consider advocacy that involves differing degrees of resistance and resistance that involves differing degrees of advocacy, and examples where the two are blurred together. For instance, rule-bending might have more of an advocacy flavour than rule-breaking but they both involve degrees of resistance.

Advocacy-resistance is typically focused on issues such as deficits in patient care, underfunding, threats to personal safety, or poor healthcare infrastructure [5,6], although it might also confront issues such as racism, sexism, and homophobia. Sometimes acts of advocacy-resistance are broadly productive and ethically unambiguous [7], such as seeking to help vulnerable individuals in systems that harbor oppression [8]. Other acts are more ambiguous, such as bending or breaking rules, noncompliance, or outright protest in seeking to help others [9,10,11]. As a result, acts of advocacy-resistance can involve conflicting values, responsibilities, and moral considerations that can in turn create ethical dilemmas both for those who engage in advocacy-resistance and for those who are called to respond [12]. That these issues are not well understood or articulated in practice only exacerbates the challenge faced by trainees in negotiating this space.

Taking a dialogic approach [13] that involved abductive group discussion and debate, reading and rereading the literature, and collaborative writing and theory building, we outlined a series of ethical dilemmas created by the pursuit of advocacy-resistance in the context of healthcare and health professions education. In this paper, we outline these dilemmas and explore their implications. Our goal in doing so was to deepen ethical thinking applied to the theory and practice of advocacy-resistance in healthcare for practitioners, trainees, teachers, and researchers in the field. We iteratively identified four broad ethical dilemmas that can confront those engaging in advocacy-resistance.

## Negotiating the locus of professional responsibility

Health professionals face dilemmas when engaging in advocacy and resistance complicates where professional responsibilities lie [14]. For instance, healthcare providers engaging in advocacy-resistance often ‘bend the rules’, use workarounds, or otherwise subvert systems and workflows to help their patients [15,16]. As an example, a physician fills out a form for a patient and “stretches the truth” in order to get them coverage for a treatment that wouldn’t otherwise be covered by their health insurance [17]. The ethical dilemma here is both about bending or breaking the rules and doing so tacitly, as the latter allows those involved to avoid dealing with conflicts between their responsibilities to ensure compliance with rules and their responsibilities to provide compassionate patient care [18].

Not all advocacy-resistance can be pursued tacitly, and this can create additional ethical dilemmas related to responsibility. For example, whistleblowing or speaking out against colleagues or superiors requires the individual concerned to balance their responsibilities to prevent harm with their responsibilities to maintain their careers and professional relationships [19]. Similarly, pushing for a treatment or policy change that goes against the political or financial interests of an organization might help one person or group, but it might also place another in greater difficulty or even in jeopardy.

Ethical dilemmas related to advocacy-resistance can become further complicated in situations where there are multiple conflicting calls on responsibility, such as to an organization or community, or to one’s colleagues or trainees. This is because advocacy-resistance involves challenging, changing, or subverting practice norms, which can have significant and unexpected consequences. This is particularly apparent when junior members of the profession (including trainees) are expected to engage in advocacy-resistance but cannot take full responsibility for their actions.

## Situating professional identity and agency within a collective

A profession is a collective and being a member of a profession involves some degree of submission to the collective’s will and values, which are typically realized through systems of accountability for its members’ actions and behaviors. Those members who fail to comply may face censure and their participation in the profession may be circumscribed or even terminated. Professional accountability covers acts of advocacy-resistance, and that can create ethical dilemmas for those involved. For instance, although an individual contemplating advocacy-resistance identifies as a member of their professional collective, they might not agree with the collective’s values and standards, particularly if they had little or no say in defining them, which means their advocacy-resistance is in part turned toward the collective will as well as to the issue at hand [20]. If advocacy-resistance is pursued on the basis of an individual’s right to freedom of speech or some other referent outside of professional propriety then that individual has stepped out of their professional role, thereby potentially exacerbating the consequences for their noncompliance and also challenging the authority of the collective in holding them accountable [21]. While individual acts of advocacy-resistance may raise challenges regarding professional identity and legitimacy, advocacy-resistance can be directly focused on the collective will, which can in turn have unpredictable and (depending on the position taken) even undesirable outcomes [22]. For instance, a 2023 draft revision to the CanMEDS competency framework sparked pushback from many members of the medical profession who were advocating for the retention of ‘medical expert’ as the core of the CanMEDS model. When the level and form of outrage spread into the public sphere it threatened to undermine the whole project [23].

The heart of the dilemma is the question of whose values should drive or constrain acts of advocacy-resistance. It can require negotiating one’s professional identity in the face of external standards and ethical imperatives that may contradict or be misaligned with those standards. It can be about exercising agency in the balance of identity and professionalism in ways that are ethically defensible even if the resulting actions break rules, challenge norms, or lead to sanctions [24]. Advocacy-resistance can therefore be understood as assertions of ethical responsibility and moral agency in the face of one’s responsibility to a professional collective in ways that may challenge the collective and one’s place within it [25].

## Balancing competing needs and priorities

Health practitioners often have to balance competing interests and priorities [20]. While dilemmas associated with responsibilities and accountabilities shape whether or not an individual engages in advocacy-resistance at all, the dilemma here is how much effort is put into advocacy-resistance relative to the effort invested in responding to other priorities [26]. For instance, the time and effort that can be safely invested in advocacy-resistance in the context of emergency or urgent patient care encounters is likely to be minimal relative to the time and effort given to advocacy-resistance in longitudinal primary care contexts even if the ethical issues are the same. Similarly, adding advocacy-resistance training or expectations may strain trainees’ time and resources, potentially detracting from their attention to other educational objectives [27]. Acuity is one modifier, complexity and inertia are others [28]. The dilemma here is not just about how to prioritize advocacy-resistance, it is also about whether the effort that can be invested is likely to achieve meaningful change. As an example, the concept of the ‘minority tax’ reflects the disproportionate advocacy-resistance load that falls to individuals advocating for their own needs in contexts that are hostile to their presence [29]. If the root of the issue at hand (difficulty, harm, injustice) is relatively intractable then practitioners may instead focus on smaller, more achievable gains that leave the deeper issues unchallenged [30].

## Responding to harm resulting from advocacy-resistance

Healthcare providers frequently make clinical decisions that impact equity of care, particularly with respect to management or distribution of limited resources. In seeking to address equity issues, providers typically engage in advocacy-resistance by going ‘above and beyond’ their core medical duties for a select few of their patients (or trainees or peers) [31]. Indeed, it is a central characteristic of advocacy-resistance that it is not applied all the time to all situations; advocacy-resistance is intrinsically selective. Ethical dilemmas may arise as providers decide who should (and by implication who should not) receive this special treatment, often by using subjective heuristics such as ‘deservingness’ [32]. For example, learners have reported being less likely to advocate for patients who were affluent, who brought their problems on themselves, or who were demanding [32].

By choosing to work on behalf of some and not others, clinicians may compound inequities rather than reduce them, and this may in turn become the focus of the advocacy-resistance of those who are less well favored. Not only can this implicit sorting create ethical dilemmas, it may inadvertently create tensions and conflicts with other values, such as treating all patients equally ‘without fear or favor’. The ‘above and beyond’ character of advocacy-resistance has the capacity to create as much, if not more, inequity than it addresses. Advocacy-resistance is all about changing or stepping outside the norms of healthcare systems. That acts of advocacy-resistance intended to help certain individuals can create more (and potentially larger) problems than those they seek to solve is not an unfamiliar dilemma in healthcare as it is encapsulated in the concept of *primum non nocere* (first do no harm). Whether this helps those pursuing advocacy-resistance is less clear.

## Discussion

Advocacy and resistance are in practice deeply intertwined, which we have reflected in our use of the dyadic concept of advocacy-resistance. This covers acts ranging from helping an individual patient to get access to services to which they are entitled to acts that challenge organizational culture and policy. Based on our arguments, advocacy-resistance can often be a zero-sum game such that, rather than being a pro-social and affirmative phenomenon (acting to improve things for others) it is a perturbing one (reconfiguring a system but not necessarily improving it). Indeed, applications of advocacy-resistance may create harm in seeking to mitigate harm, they may create inequity in seeking to reduce it, and they may place a significant load on individuals trying to negotiate this perilous landscape. That every act of advocacy-resistance intended to do good has the potential to lead to unintended harms is rarely reflected in teaching about advocacy, which tends instead to have a simplistic ‘Good Samaritan’ or heroic feel to it [33].

We would challenge this perspective and would instead argue that a more nuanced approach is needed. For instance, an approach that takes the principle of ‘first do no harm’, acknowledges that this too can create dilemmas if taken literally as harm is often required to heal, and uses this dilemma (rather than avoiding it) to help trainees and practitioners understand that while advocacy-resistance can do much good it can also be disruptive and ethically complex. Indeed, we note similarities between advocacy-resistance and the concept of entropy in that harm might be decreased locally but only at the cost of increasing it across the wider system.

Engaging in advocacy-resistance can also strain trainees’ relationships with supervisors or institutions, thereby impacting their educational experiences or future opportunities. Advocating for changes or resisting practices within healthcare settings can expose those involved to risks such as negative assessments, professional repercussions, or even jeopardizing their future career prospects. This raises concerns about whether individuals can engage in such activities safely and responsibly. One way to approach this issue is to ensure that all action is grounded in professionalism, recognizing that what it means to be professional is a concept that changes across time and context. In the case of advocacy-resistance we would argue that all such acts should be affirmative and principled (they should be for something rather than against something), deliberate (they should be undertaken intentionally and mindfully), proportionate (they should be sufficient to achieve their ends), constructive (they should be about finding and building solutions), and accountable (they should reflect professional standards), not least as a way of protecting the individual and the profession from being inhibited to the point of inaction [25]. Further, in addition to individuals making principled decisions, institutions have a responsibility to recognize they are contributing to problems around advocacy-resistance, as well. By not providing clear guidance on how advocacy-resistance should be navigated, the issue remains challenging to sort out what is an appropriate response in various situations.

In summary, every act of advocacy-resistance that is intended to reshape a system, structure, process, or practice has the potential to create ethical dilemmas, even when pursued for what is perceived to be the collective good. This is because, in changing systems, every component thereof can be stretched and pulled in new directions to compensate for the change. Both individual and collective responsibilities need to be considered in the cost-benefit analysis of any action [20,34].

**Figure 1:**
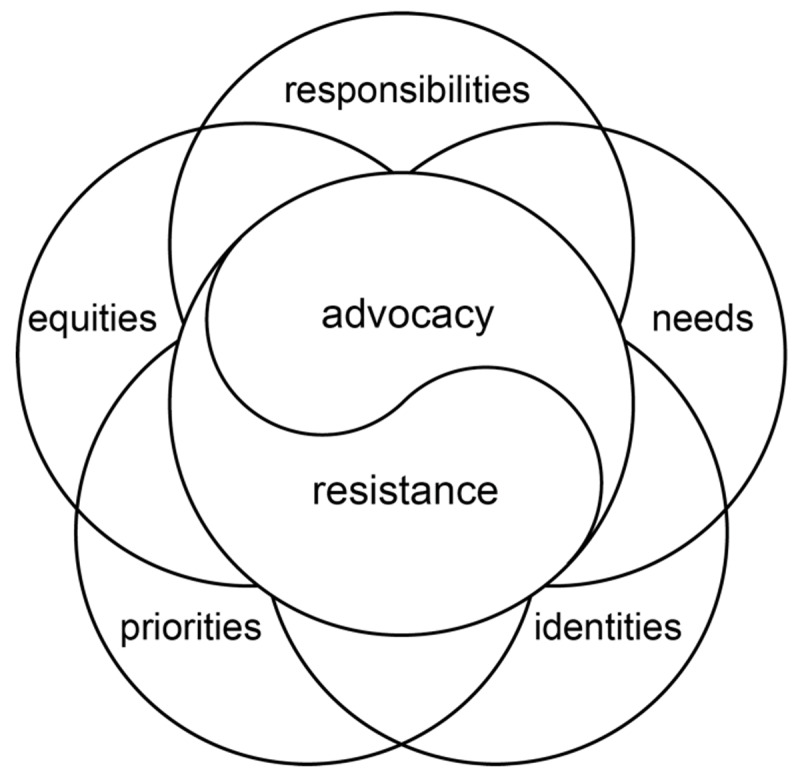
The knot of factors that shape advocacy-resistance, including the advocacy-resistance dyad itself.

The ethical dilemmas related to advocacy-resistance are not distinct; they interact with each other and in doing so they constitute a knot of ethical challenges – see Figure 1 – that need to be understood and grounded in training and practical experience. We describe this as a knot because in practice these factors cannot be easily or meaningfully separated. Navigating these ethical dilemmas requires careful consideration of principles such as beneficence, autonomy, justice, and integrity. Attention to how harms and inequities are or should be redressed is needed given that there are likely to be many differing ideas and beliefs about this. Health advocates and professionals must weigh these principles against each other and work to find ethically sound solutions that prioritize human welfare while respecting professional responsibilities and acknowledging organizational constraints.

We note several limitations to the arguments we have advanced in this paper. First, while the dialogical approach we took was, we would argue, both appropriate and proportionate to the task at hand, there is always a need for empirical work to explore the lived realities of the issues we have raised. To that end, we offer this paper as one part of an ongoing conversation about advocacy-resistance. Second, we note that the ways in which advocacy-resistance is thought about can vary according to system or jurisdiction. For instance, the discourses of advocacy-resistance vary between the United States and Canada despite having very similar medical education systems. In the United States advocacy has been promoted as a necessary responsibility of a physician (for instance by the AMA) but has had little apparent uptake in medical education [1], while in Canada, advocacy has a more explicit place in medical education through being one of the seven CanMEDS roles around which programs are articulated [33]. Interestingly, resistance has rarely been considered in medical education in either country [35]. Advocacy is also promoted in medical education in the Netherlands [36], the United Kingdom [37], and in many other systems around the world. Although the underlying dynamics of advocacy-resistance are common to many (if not all) healthcare contexts, the way they are realized can vary according to many contextual factors and this should be borne in mind when translating our arguments into practice.

A third issue reflects the changing nature of advocacy-resistance in medical education and practice. There has been a noticeable increase in attention to and participation in advocacy-resistance in recent years, in great part around issues of diversity, equity, inclusion and anti-racism, with many positions being taken on these matters. Our arguments in this paper reflect the current state of the art, but as this continues to change, the currency of the paper is also likely to change, likely in unexpected ways.

In conclusion, while advocacy-resistance are often thought to be courageous and pro-social acts that move our systems to do better, there are important ethical dilemmas that need to be considered. Like everything else in medical education, every act and every decision has potential consequences and downstream effects. This is not to say that individuals should not engage in advocacy-resistance, only that they operate in an ecosystem that must be considered before acting.

## Disclaimer

This work was prepared by a civilian employee of the US Government as part of the individual’s official duties and therefore is in the public domain. The opinions and assertions expressed herein are those of the author(s) and do not necessarily reflect the official policy or position of the Uniformed Services University or the Department of Defense.
